# 1-(4-Meth­oxy­phen­yl)-4-(4-methyl­phen­yl)-3-phen­oxy­azetidin-2-one

**DOI:** 10.1107/S1600536811000663

**Published:** 2011-01-12

**Authors:** Mehmet Akkurt, Yılmaz Dağdemir, Aliasghar Jarrahpour, Maryam Rostami, Orhan Büyükgüngör

**Affiliations:** a Department of Physics, Faculty of Sciences, Erciyes University, 38039 Kayseri, Turkey; b Department of Chemistry, College of Sciences, Shiraz University, 71454 Shiraz, Iran; c Department of Physics, Faculty of Arts and Sciences, Ondokuz Mayıs University, 55139 Samsun, Turkey

## Abstract

The central β-lactam ring of the title compound, C_23_H_21_NO_3_, is almost planar (r.m.s. deviation = 0.032Å). The meth­oxy­benzene ring is almost coplanar with the β-lactam ring [dihedral angle = 1.87 (11)°], whereas the tolyl ring is almost normal to it [75.73 (12)°]. The dihedral angle between the β-lactam ring and the O-bonded phenyl ring is 51.95 (12)°. An intra­molecular C—H⋯O inter­action generates an *S*(6) ring. The crystal structure features inter­molecular C—H⋯O hydrogen bonds, forming layers parallel to (011), and weak C—H⋯π inter­actions. Two aromatic π–π stacking inter­actions [centroid–centroid distances = 3.6744 (12) and 3.6799 (11) Å] are also observed.

## Related literature

For the synthesis of the title compound and background to the biological properties of β-lactam compounds, see: Jarrahpour & Zarei (2010 ▶). For bond-length data, see: Allen *et al.* (1987 ▶). For hydrogen-bond motifs, see: Bernstein *et al.* (1995 ▶).
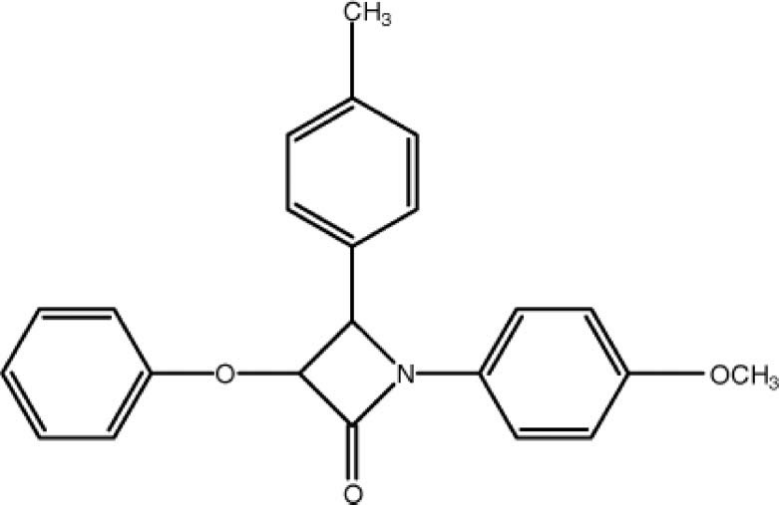


## Experimental

### 

#### Crystal data

C_23_H_21_NO_3_
*M* *_r_* = 359.41
Triclinic, 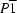

*a* = 6.0764 (3) Å
*b* = 9.9545 (5) Å
*c* = 16.4519 (10) Å
α = 104.360 (4)°
β = 91.261 (5)°
γ = 97.724 (4)°
*V* = 953.71 (9) Å^3^
*Z* = 2
Mo *K*α radiation
μ = 0.08 mm^−1^
*T* = 296 K
0.60 × 0.37 × 0.12 mm

#### Data collection

Stoe IPDS 2 diffractometer
Absorption correction: integration (*X-RED32*; Stoe & Cie, 2002 ▶) *T*_min_ = 0.964, *T*_max_ = 0.990
13748 measured reflections
3961 independent reflections
2608 reflections with *I* > 2σ(*I*)
*R*_int_ = 0.055

#### Refinement

*R*[*F* ^2^ > 2σ(*F* ^2^)] = 0.048
*wR*(*F* ^2^) = 0.119
*S* = 1.03
3961 reflections
246 parameters
H-atom parameters constrained
Δρ_max_ = 0.13 e Å^−3^
Δρ_min_ = −0.17 e Å^−3^

### 

Data collection: *X-AREA* (Stoe & Cie, 2002 ▶); cell refinement: *X-AREA*; data reduction: *X-RED32* (Stoe & Cie, 2002 ▶); program(s) used to solve structure: *SIR97* (Altomare *et al.*, 1999 ▶); program(s) used to refine structure: *SHELXL97* (Sheldrick, 2008 ▶); molecular graphics: *ORTEP-3* (Farrugia, 1997 ▶); software used to prepare material for publication: *WinGX* (Farrugia, 1999 ▶).

## Supplementary Material

Crystal structure: contains datablocks global, I. DOI: 10.1107/S1600536811000663/hb5783sup1.cif

Structure factors: contains datablocks I. DOI: 10.1107/S1600536811000663/hb5783Isup2.hkl

Additional supplementary materials:  crystallographic information; 3D view; checkCIF report

## Figures and Tables

**Table 1:** Hydrogen-bond geometry (Å, °) *Cg*4 is the centroid of the C17–C22 benzene ring.

*D*—H⋯*A*	*D*—H	H⋯*A*	*D*⋯*A*	*D*—H⋯*A*
C18—H18⋯O2	0.93	2.47	3.093 (2)	125
C9—H9⋯O2^i^	0.98	2.51	3.446 (2)	160
C15—H15⋯O1^i^	0.93	2.54	3.435 (2)	162
C19—H19⋯O2^ii^	0.93	2.54	3.415 (2)	156
C23—H23*C*⋯O2^iii^	0.96	2.52	3.184 (2)	126
C5—H5⋯*Cg*4^iv^	0.93	2.96	3.544 (3)	122

Symmetry codes: (i) 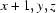; (ii) 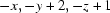; (iii) 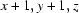; (iv) 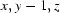.

**Fig. 1.**
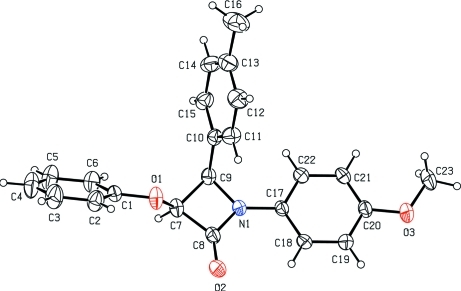
The title compound with displacement ellipsoids for non-H atoms drawn at the 30% probability level.

**Fig. 2.**
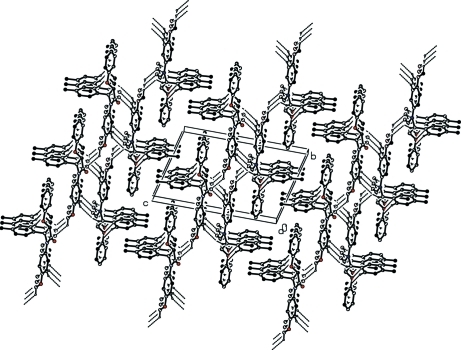
View of the packing of (I) down the a axis. All H atoms are omitted for clarity.

## supplementary crystallographic information

### Comment

The title compound, (I), is a β-lactam derivative with potential biological properties (Jarrahpour & Zarei, 2010); we now describe its crystal structure. In the title compound (I), (Fig. 1), the β-lactam ring (N1/C7–C9) is nearly planar [r.m.s. deviation = 0.032Å]. The dihedral angles between the planes of the rings in (I) are given in Table 2. The molecular dimensions are normal and lie within expected values for corresponding bond distances and angles (Allen *et al.*, 1987).

The molecular structure is stabilized by intramolecular C—H···O hydrogen bonds, forming an S(6) graph-set motif (Bernstein *et al.*, 1995) (Table 1). In the crystal structure, molecules are linked *via* intermolecular C—H···O hydrogen bonds (Table 1, Fig. 2), forming layers parallel to the *bc* plane. In addition, C—H···π interactions and two π-π stacking interactions [*Cg*1···*Cg*3(*x*, *y*, *z*) = 3.6744 (12) Å and *Cg*4···*Cg*4(1 - *x*, 2 - *y*, 1 - *z*) = 3.6799 (11) Å, where *Cg*1, *Cg*3 and *Cg*4 are the centroids of the N1/C7–C9 β-lactam, the C10–C15 and C17–C22 benzene rings, respectively] contribute to the stabilization of the structure.

### Experimental

The title compound was prepared as described by Jarrahpour & Zarei (2010) and colourless prisms of (I) were recrystallized from ethyl acetate.

### Refinement

All H atoms were placed at calculated positions and were treated as riding on their parent atoms with C—H = 0.93 (aromatic), 0.96(methyl) and 0.98 Å (methine), and with *U*_iso_(H) = 1.5*U*_eq_(C) for methyl and *U*_iso_(H) = 1.2*U*_eq_(C) for aromatic, methine.

### Crystal data

**Table:**

C_23_H_21_NO_3_	*Z* = 2
*M**_r_* = 359.41	*F*(000) = 380
Triclinic, *P*1	*D*_x_ = 1.252 Mg m^−^^3^
Hall symbol: -P 1	Mo *K*α radiation, λ = 0.71073 Å
*a* = 6.0764 (3) Å	Cell parameters from 19074 reflections
*b* = 9.9545 (5) Å	θ = 2.1–27.6°
*c* = 16.4519 (10) Å	µ = 0.08 mm^−^^1^
α = 104.360 (4)°	*T* = 296 K
β = 91.261 (5)°	Prism, colourless
γ = 97.724 (4)°	0.60 × 0.37 × 0.12 mm
*V* = 953.71 (9) Å^3^	

### Data collection

**Table:**

Stoe IPDS 2 diffractometer	3961 independent reflections
Radiation source: sealed X-ray tube, 12 x 0.4 mm long-fine focus	2608 reflections with *I* > 2σ(*I*)
plane graphite	*R*_int_ = 0.055
Detector resolution: 6.67 pixels mm^-1^	θ_max_ = 26.5°, θ_min_ = 2.1°
ω scans	*h* = −7→7
Absorption correction: integration (*X-RED32*; Stoe & Cie, 2002)	*k* = −12→12
*T*_min_ = 0.964, *T*_max_ = 0.990	*l* = −20→20
13748 measured reflections	

### Refinement

**Table:**

Refinement on *F*^2^	Primary atom site location: structure-invariant direct methods
Least-squares matrix: full	Secondary atom site location: difference Fourier map
*R*[*F*^2^ > 2σ(*F*^2^)] = 0.048	Hydrogen site location: inferred from neighbouring sites
*wR*(*F*^2^) = 0.119	H-atom parameters constrained
*S* = 1.03	*w* = 1/[σ^2^(*F*_o_^2^) + (0.0548*P*)^2^ + 0.0685*P*] where *P* = (*F*_o_^2^ + 2*F*_c_^2^)/3
3961 reflections	(Δ/σ)_max_ < 0.001
246 parameters	Δρ_max_ = 0.13 e Å^−^^3^
0 restraints	Δρ_min_ = −0.17 e Å^−^^3^

### Special details

**Table:**

Geometry. Bond distances, angles *etc*. have been calculated using the rounded fractional coordinates. All su's are estimated from the variances of the (full) variance-covariance matrix. The cell e.s.d.'s are taken into account in the estimation of distances, angles and torsion angles
Refinement. Refinement on *F*^2^ for ALL reflections except those flagged by the user for potential systematic errors. Weighted *R*-factors *wR* and all goodnesses of fit *S* are based on *F*^2^, conventional *R*-factors *R* are based on *F*, with *F* set to zero for negative *F*^2^. The observed criterion of *F*^2^ > σ(*F*^2^) is used only for calculating -*R*-factor-obs *etc*. and is not relevant to the choice of reflections for refinement. *R*-factors based on *F*^2^ are statistically about twice as large as those based on *F*, and *R*-factors based on ALL data will be even larger.

### Fractional atomic coordinates and isotropic or equivalent isotropic displacement parameters (Å^2^)

**Table:**

	*x*	*y*	*z*	*U*_iso_*/*U*_eq_	
O1	0.1189 (2)	0.46929 (12)	0.24918 (8)	0.0593 (4)	
O2	−0.0121 (2)	0.69814 (13)	0.39817 (9)	0.0661 (5)	
O3	0.5557 (2)	1.34079 (13)	0.45143 (10)	0.0691 (5)	
N1	0.3435 (2)	0.76262 (13)	0.35417 (9)	0.0449 (4)	
C1	0.0980 (3)	0.32558 (17)	0.22621 (11)	0.0466 (5)	
C2	−0.0981 (3)	0.2579 (2)	0.18159 (14)	0.0659 (7)	
C3	−0.1326 (4)	0.1146 (2)	0.15480 (17)	0.0855 (9)	
C4	0.0243 (5)	0.0375 (2)	0.17266 (18)	0.0906 (10)	
C5	0.2170 (5)	0.1043 (2)	0.21619 (16)	0.0841 (10)	
C6	0.2565 (3)	0.2490 (2)	0.24332 (13)	0.0626 (7)	
C7	0.2563 (3)	0.54632 (17)	0.32009 (11)	0.0493 (6)	
C8	0.1608 (3)	0.67635 (17)	0.36523 (11)	0.0481 (6)	
C9	0.4601 (3)	0.65118 (17)	0.30514 (11)	0.0456 (5)	
C10	0.5040 (3)	0.66017 (17)	0.21748 (11)	0.0461 (5)	
C11	0.3614 (3)	0.7126 (2)	0.17006 (12)	0.0590 (7)	
C12	0.4120 (4)	0.7251 (2)	0.09059 (14)	0.0725 (8)	
C13	0.6024 (4)	0.6850 (2)	0.05514 (13)	0.0711 (8)	
C14	0.7428 (3)	0.6312 (2)	0.10147 (15)	0.0733 (8)	
C15	0.6962 (3)	0.6196 (2)	0.18176 (13)	0.0603 (7)	
C16	0.6603 (5)	0.7014 (3)	−0.03125 (17)	0.1131 (13)	
C17	0.4029 (2)	0.90918 (16)	0.37839 (10)	0.0417 (5)	
C18	0.2574 (3)	0.99309 (17)	0.42190 (11)	0.0489 (6)	
C19	0.3149 (3)	1.13583 (18)	0.44533 (12)	0.0523 (6)	
C20	0.5185 (3)	1.19731 (17)	0.42538 (11)	0.0494 (6)	
C21	0.6635 (3)	1.11455 (18)	0.38189 (12)	0.0514 (6)	
C22	0.6062 (3)	0.97064 (18)	0.35878 (11)	0.0488 (6)	
C23	0.7547 (3)	1.4105 (2)	0.42925 (15)	0.0691 (7)	
H2	−0.20600	0.30950	0.16980	0.0790*	
H3	−0.26370	0.06920	0.12420	0.1030*	
H4	−0.00100	−0.05980	0.15510	0.1090*	
H5	0.32400	0.05210	0.22790	0.1010*	
H6	0.38930	0.29390	0.27290	0.0750*	
H7	0.29430	0.48910	0.35750	0.0590*	
H9	0.59590	0.64290	0.33570	0.0550*	
H11	0.23000	0.73970	0.19210	0.0710*	
H12	0.31430	0.76160	0.06040	0.0870*	
H14	0.87190	0.60190	0.07840	0.0880*	
H15	0.79530	0.58410	0.21190	0.0720*	
H16A	0.77970	0.77730	−0.02580	0.1360*	
H16B	0.53250	0.72090	−0.05910	0.1360*	
H16C	0.70560	0.61630	−0.06360	0.1360*	
H18	0.12050	0.95250	0.43520	0.0590*	
H19	0.21710	1.19170	0.47470	0.0630*	
H21	0.79960	1.15550	0.36810	0.0620*	
H22	0.70480	0.91470	0.32990	0.0590*	
H23A	0.75830	1.38950	0.36910	0.0830*	
H23B	0.87980	1.37960	0.45200	0.0830*	
H23C	0.76110	1.50980	0.45150	0.0830*	

### Atomic displacement parameters (Å^2^)

**Table:**

	*U*^11^	*U*^22^	*U*^33^	*U*^12^	*U*^13^	*U*^23^
O1	0.0676 (7)	0.0352 (7)	0.0707 (9)	−0.0008 (5)	−0.0211 (6)	0.0116 (6)
O2	0.0539 (7)	0.0533 (8)	0.0853 (10)	−0.0075 (6)	0.0196 (7)	0.0132 (7)
O3	0.0758 (8)	0.0372 (7)	0.0866 (10)	−0.0080 (6)	0.0139 (7)	0.0091 (6)
N1	0.0449 (7)	0.0359 (7)	0.0502 (9)	−0.0019 (6)	0.0055 (6)	0.0079 (6)
C1	0.0499 (9)	0.0379 (9)	0.0504 (10)	0.0010 (7)	0.0033 (7)	0.0112 (8)
C2	0.0555 (10)	0.0520 (12)	0.0820 (15)	0.0035 (9)	−0.0070 (10)	0.0048 (10)
C3	0.0791 (14)	0.0564 (14)	0.102 (2)	−0.0123 (11)	−0.0153 (13)	−0.0020 (12)
C4	0.125 (2)	0.0410 (12)	0.096 (2)	0.0026 (13)	−0.0111 (16)	0.0054 (12)
C5	0.1158 (19)	0.0507 (13)	0.0853 (17)	0.0252 (12)	−0.0183 (14)	0.0114 (12)
C6	0.0673 (11)	0.0499 (11)	0.0688 (13)	0.0097 (9)	−0.0111 (10)	0.0125 (10)
C7	0.0540 (9)	0.0372 (9)	0.0547 (11)	−0.0006 (7)	−0.0069 (8)	0.0125 (8)
C8	0.0474 (9)	0.0409 (9)	0.0536 (11)	−0.0043 (7)	0.0018 (8)	0.0135 (8)
C9	0.0438 (8)	0.0399 (9)	0.0516 (10)	0.0048 (7)	−0.0029 (7)	0.0099 (8)
C10	0.0442 (8)	0.0394 (9)	0.0514 (11)	0.0043 (7)	0.0003 (7)	0.0064 (8)
C11	0.0564 (10)	0.0684 (13)	0.0573 (12)	0.0190 (9)	0.0046 (9)	0.0198 (10)
C12	0.0798 (14)	0.0818 (16)	0.0598 (13)	0.0099 (11)	−0.0018 (11)	0.0267 (11)
C13	0.0748 (13)	0.0746 (15)	0.0527 (13)	−0.0134 (11)	0.0052 (10)	0.0081 (11)
C14	0.0585 (11)	0.0793 (15)	0.0693 (15)	0.0020 (10)	0.0201 (11)	−0.0022 (12)
C15	0.0498 (9)	0.0588 (12)	0.0684 (14)	0.0119 (8)	0.0036 (9)	0.0067 (10)
C16	0.127 (2)	0.132 (3)	0.0654 (17)	−0.0295 (19)	0.0188 (15)	0.0213 (16)
C17	0.0442 (8)	0.0363 (9)	0.0422 (9)	−0.0027 (6)	0.0009 (7)	0.0099 (7)
C18	0.0446 (8)	0.0454 (10)	0.0540 (11)	−0.0025 (7)	0.0118 (7)	0.0116 (8)
C19	0.0547 (9)	0.0424 (10)	0.0568 (11)	0.0033 (8)	0.0126 (8)	0.0080 (8)
C20	0.0562 (9)	0.0366 (9)	0.0517 (11)	−0.0049 (7)	0.0019 (8)	0.0103 (8)
C21	0.0437 (8)	0.0464 (10)	0.0613 (12)	−0.0075 (7)	0.0066 (8)	0.0157 (8)
C22	0.0427 (8)	0.0457 (10)	0.0552 (11)	0.0008 (7)	0.0075 (7)	0.0103 (8)
C23	0.0711 (12)	0.0471 (11)	0.0829 (15)	−0.0178 (9)	−0.0034 (10)	0.0198 (10)

### Geometric parameters (Å, °)

**Table:**

O1—C1	1.373 (2)	C18—C19	1.371 (3)
O1—C7	1.410 (2)	C19—C20	1.388 (3)
O2—C8	1.214 (2)	C20—C21	1.378 (3)
O3—C20	1.371 (2)	C21—C22	1.381 (3)
O3—C23	1.413 (2)	C2—H2	0.9300
N1—C8	1.354 (2)	C3—H3	0.9300
N1—C9	1.475 (2)	C4—H4	0.9300
N1—C17	1.408 (2)	C5—H5	0.9300
C1—C2	1.383 (3)	C6—H6	0.9300
C1—C6	1.371 (3)	C7—H7	0.9800
C2—C3	1.370 (3)	C9—H9	0.9800
C3—C4	1.371 (4)	C11—H11	0.9300
C4—C5	1.358 (4)	C12—H12	0.9300
C5—C6	1.384 (3)	C14—H14	0.9300
C7—C8	1.519 (3)	C15—H15	0.9300
C7—C9	1.574 (3)	C16—H16A	0.9600
C9—C10	1.495 (2)	C16—H16B	0.9600
C10—C11	1.388 (3)	C16—H16C	0.9600
C10—C15	1.383 (3)	C18—H18	0.9300
C11—C12	1.381 (3)	C19—H19	0.9300
C12—C13	1.370 (3)	C21—H21	0.9300
C13—C14	1.376 (3)	C22—H22	0.9300
C13—C16	1.514 (3)	C23—H23A	0.9600
C14—C15	1.386 (3)	C23—H23B	0.9600
C17—C18	1.386 (2)	C23—H23C	0.9600
C17—C22	1.385 (2)		
			
C1—O1—C7	120.21 (14)	C3—C2—H2	120.00
C20—O3—C23	117.71 (15)	C2—C3—H3	120.00
C8—N1—C9	96.00 (13)	C4—C3—H3	120.00
C8—N1—C17	132.78 (13)	C3—C4—H4	120.00
C9—N1—C17	131.20 (13)	C5—C4—H4	120.00
O1—C1—C2	115.45 (16)	C4—C5—H5	120.00
O1—C1—C6	124.67 (17)	C6—C5—H5	120.00
C2—C1—C6	119.87 (17)	C1—C6—H6	120.00
C1—C2—C3	119.70 (19)	C5—C6—H6	120.00
C2—C3—C4	120.6 (2)	O1—C7—H7	113.00
C3—C4—C5	119.5 (2)	C8—C7—H7	113.00
C4—C5—C6	120.9 (2)	C9—C7—H7	113.00
C1—C6—C5	119.4 (2)	N1—C9—H9	112.00
O1—C7—C8	111.20 (14)	C7—C9—H9	112.00
O1—C7—C9	117.37 (14)	C10—C9—H9	111.00
C8—C7—C9	85.70 (13)	C10—C11—H11	120.00
O2—C8—N1	132.64 (17)	C12—C11—H11	120.00
O2—C8—C7	134.97 (17)	C11—C12—H12	119.00
N1—C8—C7	92.39 (14)	C13—C12—H12	119.00
N1—C9—C7	85.80 (12)	C13—C14—H14	119.00
N1—C9—C10	115.03 (14)	C15—C14—H14	119.00
C7—C9—C10	119.15 (15)	C10—C15—H15	120.00
C9—C10—C11	122.47 (16)	C14—C15—H15	120.00
C9—C10—C15	119.98 (17)	C13—C16—H16A	109.00
C11—C10—C15	117.52 (17)	C13—C16—H16B	109.00
C10—C11—C12	120.93 (18)	C13—C16—H16C	109.00
C11—C12—C13	121.6 (2)	H16A—C16—H16B	110.00
C12—C13—C14	117.7 (2)	H16A—C16—H16C	109.00
C12—C13—C16	121.6 (2)	H16B—C16—H16C	109.00
C14—C13—C16	120.7 (2)	C17—C18—H18	120.00
C13—C14—C15	121.53 (19)	C19—C18—H18	120.00
C10—C15—C14	120.74 (18)	C18—C19—H19	120.00
N1—C17—C18	120.09 (13)	C20—C19—H19	120.00
N1—C17—C22	120.40 (14)	C20—C21—H21	120.00
C18—C17—C22	119.52 (16)	C22—C21—H21	120.00
C17—C18—C19	120.12 (16)	C17—C22—H22	120.00
C18—C19—C20	120.29 (17)	C21—C22—H22	120.00
O3—C20—C19	114.80 (16)	O3—C23—H23A	109.00
O3—C20—C21	125.32 (16)	O3—C23—H23B	109.00
C19—C20—C21	119.88 (17)	O3—C23—H23C	109.00
C20—C21—C22	119.83 (17)	H23A—C23—H23B	109.00
C17—C22—C21	120.36 (16)	H23A—C23—H23C	109.00
C1—C2—H2	120.00	H23B—C23—H23C	109.00
			
C7—O1—C1—C2	154.15 (17)	O1—C7—C8—N1	−120.21 (15)
C7—O1—C1—C6	−26.9 (3)	C9—C7—C8—O2	176.8 (2)
C1—O1—C7—C8	−145.56 (15)	O1—C7—C9—C10	−2.5 (2)
C1—O1—C7—C9	118.10 (17)	C8—C7—C9—N1	2.27 (12)
C23—O3—C20—C21	2.6 (3)	O1—C7—C8—O2	59.0 (3)
C23—O3—C20—C19	−176.70 (17)	N1—C9—C10—C11	−32.4 (2)
C8—N1—C9—C7	−2.55 (13)	N1—C9—C10—C15	145.45 (17)
C17—N1—C8—O2	1.7 (3)	C7—C9—C10—C11	67.2 (2)
C9—N1—C8—O2	−176.6 (2)	C7—C9—C10—C15	−114.9 (2)
C9—N1—C8—C7	2.64 (14)	C9—C10—C15—C14	−178.05 (18)
C17—N1—C8—C7	−179.03 (17)	C15—C10—C11—C12	−0.8 (3)
C17—N1—C9—C7	179.07 (16)	C9—C10—C11—C12	177.14 (18)
C9—N1—C17—C18	177.01 (16)	C11—C10—C15—C14	−0.1 (3)
C8—N1—C17—C22	179.43 (17)	C10—C11—C12—C13	0.8 (3)
C9—N1—C17—C22	−2.8 (3)	C11—C12—C13—C14	0.1 (3)
C8—N1—C17—C18	−0.8 (3)	C11—C12—C13—C16	−178.7 (2)
C8—N1—C9—C10	117.76 (16)	C12—C13—C14—C15	−1.0 (3)
C17—N1—C9—C10	−60.6 (2)	C16—C13—C14—C15	177.9 (2)
O1—C1—C6—C5	−179.42 (19)	C13—C14—C15—C10	1.0 (3)
O1—C1—C2—C3	178.99 (19)	N1—C17—C22—C21	179.40 (16)
C6—C1—C2—C3	0.0 (3)	C18—C17—C22—C21	−0.4 (3)
C2—C1—C6—C5	−0.6 (3)	N1—C17—C18—C19	−179.84 (16)
C1—C2—C3—C4	0.8 (4)	C22—C17—C18—C19	−0.1 (3)
C2—C3—C4—C5	−1.0 (4)	C17—C18—C19—C20	0.3 (3)
C3—C4—C5—C6	0.5 (4)	C18—C19—C20—C21	0.0 (3)
C4—C5—C6—C1	0.3 (4)	C18—C19—C20—O3	179.34 (17)
O1—C7—C9—N1	113.95 (15)	O3—C20—C21—C22	−179.72 (17)
C8—C7—C9—C10	−114.14 (17)	C19—C20—C21—C22	−0.4 (3)
C9—C7—C8—N1	−2.47 (13)	C20—C21—C22—C17	0.6 (3)

### Hydrogen-bond geometry (Å, °)

**Table:**

Cg4 is the centroid of the C17–C22 benzene ring.

**Table:**

*D*—H···*A*	*D*—H	H···*A*	*D*···*A*	*D*—H···*A*
C18—H18···O2	0.93	2.47	3.093 (2)	125
C9—H9···O2^i^	0.98	2.51	3.446 (2)	160
C15—H15···O1^i^	0.93	2.54	3.435 (2)	162
C19—H19···O2^ii^	0.93	2.54	3.415 (2)	156
C23—H23C···O2^iii^	0.96	2.52	3.184 (2)	126
C5—H5···Cg4^iv^	0.93	2.96	3.544 (3)	122

Symmetry codes: (i) *x*+1, *y*, *z*; (ii) −*x*, −*y*+2, −*z*+1; (iii) *x*+1, *y*+1, *z*; (iv) *x*, *y*−1, *z*.

### Table 2 The dihedral angles between the mean planes of the rings in (I) (°)

**Table:**

	Ring-2	Ring-3	Ring-4
Ring-1	51.95 (12)	75.73 (12)	1.87 (11)
Ring-2		86.93 (11)	50.10 (10)
Ring-3			76.54 (9)

Ring-1 : N1/C7–C9 β-lactam ring, Ring-2 : C1–C6 phenyl ring, Ring-3 : C10–C15 benzene ring, Ring-4 : C17–C22 benzene ring.
